# Antibiofilm Effects of Heated Scallop Shell Powder on *Campylobacter jejuni* Biofilms

**DOI:** 10.3390/membranes12010043

**Published:** 2021-12-29

**Authors:** Haruka Tsukuda, Taiki Akimoto, Nona Fukikoshi, Resei Wada, Jun Sawai

**Affiliations:** 1 Department of Nutrition and Life Science, Faculty of Health and Medical Sciences, Kanagawa Institute of Technology, 1030 Shimo-Ogino, Atsugi 243-0292, Kanagawa, Japan; tsukuda_ha@yahoo.co.jp (H.T.); lifescience.autumbook@gmail.com (T.A.); nona.fukkoshi069@gmail.com (N.F.); 2 Faculty of Applied Bioscience, Kanagawa Institute of Technology, 1030 Shimo-Ogino, Atsugi 243-0292, Kanagawa, Japan; wada@bio.kanagawa-it.ac.jp

**Keywords:** heated scallop shell powder, calcium oxide, biofilm, *Campylobacter jejuni*, antibiofilm activity, antibacterial activity

## Abstract

Methods to reuse large numbers of scallop shells from the harvesting regions of Japan are being explored. The major component of scallop shells is calcium carbonate (CaCO_3_), which forms the powerful bactericidal agent, calcium oxide (CaO), when heated. Heated scallop shell powder (HSSP) exhibits strong and broad-spectrum antimicrobial activity against bacteria, fungi, and viruses. This study investigated the antibiofilm activity of HSSP against the biofilms of *Campylobacter jejuni*, which is the predominant species in campylobacteriosis. Biofilm samples of *C. jejuni* were prepared on 0.45 µm filter paper under microaerobic conditions. The HSSP treatment inactivated and eradicated *C. jejuni* biofilms. The resistance of *C. jejuni* biofilms to HSSP was significantly higher than that of the floating cells. Moreover, the antibiofilm activity of the HSSP treatment against *C. jejuni* biofilms was higher than that of NaOH treatment at the same pH. These results indicated that HSSP treatment is an effective method for controlling *C. jejuni* biofilms.

## 1. Introduction

*Campylobacter jejuni* is a Gram-negative, curved-to-spiral rod with polar flagella that grows best in a microaerophilic environment ranging from 37 °C to 42 °C [1]. Campylobacteriosis has been the most common cause of bacterial food poisoning in Japan for more than a decade, and *C. jejuni* is the predominant species responsible for campylobacteriosis (Japan’s Ministry of Health, Labor, and Welfare statistical survey for 2020). *C. jejuni* can form biofilms [2] that can protect microorganisms from environmental stress and antimicrobial agents, and some studies have indicated that bacteria in biofilms are as much as 1000 times more resistant to antibiotics than planktonic cells [3,4,5].

Large numbers of scallop shells are heaped on the shore in harvesting regions, creating problems such as offensive odors and soil pollution due to the leaching of heavy metals from the viscera [6,7]. The major component of scallop shells is calcium carbonate (CaCO_3_), which forms the powerful bactericidal agent, calcium oxide (CaO), when heated. Heated scallop shell powder (HSSP) exhibits strong and broad-spectrum antimicrobial activity against bacteria [8,9,10], fungi [11], and viruses [12,13]. The increase in the use of HSSP in food processing can contribute to reducing pollution problems because waste shells can be a useful resource. Several studies have reported that HSSP treatment is effective for the disinfection and preservation of foods such as fresh vegetables [14,15,16], frankfurters [17], chicken [18], frozen meat [10], and fresh fish [19]. 

Although HSSP is effective for disinfecting food, it also generates CaCO_3_ scales on the surfaces of processing equipment because of the reaction of CaO with CO_2_ in the air. We showed that the addition of sorbitol suppressed the scale generation in the processing equipment [20]. Furthermore, we prepared sorbitol-coated HSSP, resulting in improved flowability and handling qualities, reduced scale buildup, and improved hygroscopicity [21]. In recent years, HSSP has been applied in the medical field, and it has been confirmed that HSSP is effective in disinfecting wounds [22] and that ointments containing HSSP [23] are effective.

HSSP treatment can kill and eradicate floating bacterial cells as well as cells in the biofilm state, and there are reports on the antibiofilm activities of HSSP treatment against *Salmonella*, *Escherichia coli*, *Staphylococcus aureus*, and *Listeria* spp. [17,24,25,26,27]. However, only a few studies have reported the effect of HSSP treatment on *Campylobacter* biofilms. Therefore, this study aimed to explore the effects of HSSP treatment on *C. jejuni* biofilms. In recent years, superior materials with antimicrobial activity, such as silver nanoparticles [28,29] and graphene oxide [30,31], have been reported, but these materials cannot be added directly to food. Contrastingly, HSSP is approved as a food additive in Japan, and it provides calcium supplementation in addition to improved shelf life owing to its antimicrobial activity. If its use is further expanded, it could be a clue to solving the pollution problem caused by the seashells, as mentioned above.

## 2. Materials and Methods

### 2.1. Test Bacteria

*C. jejuni* sp. *jejuni* ATCC 29428 was used as a reference strain. This was spread onto a Campylobacter blood-free selective medium agar (charcoal cefoperazone deoxycholate agar [CCDA]; Oxiid, Cambridge, UK) plate and incubated at 37 °C for 48 h under microaerobic conditions using AneroPack MicroAnero (Mstsubishi Gas Chemical Co., Tokyo, Japan). A pure colony that formed on the CCDA plate was picked and inoculated into Bolton selective enrichment broth and precultured for 24 h at 37 °C under microaerobic conditions.

### 2.2. Preparation of Biofilm Sample

The *C. jejuni* biofilm sample was prepared by partially modifying the procedure described by Lu et al. [32]. The preculture was diluted with saline, inoculated onto CCDA plates at approximately 10^1^ colony-forming units (CFU)/plate, and incubated for 48 h at 37 °C under microaerobic conditions to form colonies. Sterilized nitrocellulose filter paper (pore size, 0.45 μm; diameter, 47 mm; Sartorius, Göttingen, Germany) was cut into equal parts. This filter paper was placed on the colony formed on the CCDA plate and further incubated under the same conditions for 48 h. After incubation, the color of the filter paper on the colony changed from white to brown and the formation of *C. jejuni* biofilms on the filter paper was observed. Filter paper with biofilms was used as the sample for the subsequent HSSP treatment. 

The biofilm samples prepared in this study were examined using a scanning electron microscope (SEM; Phenom G2Pro, Thermo Fisher Scientific, Waltham, MA, USA) in a low-vacuum mode without covering them with gold or copper.

### 2.3. HSSP Treatment

#### 2.3.1. Preparation of HSSP

Scallop shell powder (*Patinopecten yessoensis*) was obtained from Soycom Co., Ltd. (Atsugi, Japan). The powder was heated in an electric oven (F-120-SP; Tokyo Garasu Kikai Co. Ltd., Tokyo, Japan) for 1 h at 1000 °C and then ground in a ball mill. The mean particle size of HSSP was approximately 5 µm after grinding and was stored in a desiccator before use. Sterile physiological saline (0.85% NaCl; FUJIFILM Wako Chemicals Co. Ltd., Osaka, Japan) was added to the HSSP to prepare a slurry.

#### 2.3.2. Floating Cells of *C. jejuni*

The HSSP slurry (20 mL) was poured into a glass vial (inner diameter: 32 mm, height: 75 mm, Tokyo Garasu Kikai Co. Ltd.). A preculture of *C. jejuni* (0.2 mL) was added to the HSSP slurry and stirred with a magnetic stirrer, after which the experiment was started. The initial bacterial concentration was 10^5^–10^6^ CFU/mL. A portion of the slurry (0.1 mL) was sampled at a specified time, diluted with sterile physiological saline, and incubated on a CCDA plate under microaerobic conditions (37 °C, 48 h). After incubation, the number of colonies that formed on the plates was counted to determine the number of viable bacterial cells.

#### 2.3.3. Biofilms of *C. jejuni*

An HSSP slurry (20 mL) was prepared at a specified concentration and added to a sterile plastic Petri dish with an inner diameter of 90 mm (Sansei Medical Co. Ltd., Kyoto, Japan). The biofilm sample was immersed in the HSSP slurry, which was then removed and placed in a stomacher bag (PHXON-20, ELMEX Co. Ltd., Tokyo, Japan) containing 10 mL of soybean casein digested lecithin polysorbate 80 medium (Eiken Chemicals, Tokyo, Japan). Next, the cells were subjected to stomacher treatment (1 min) to collect bacterial cells. A portion of the solution subjected to stomacher treatment was applied to CCDA plates under microaerobic conditions (37 °C, 48 h) to count the number of viable bacterial cells.

### 2.4. Statistical Analysis

All experiments were performed in triplicate (*n* = 3). The obtained data were analyzed by analysis of variance (Tukey’s method) in the Bell Curve for Excel version 2.0.3 (Social Survey Research Information Co., Ltd., Tokyo, Japan) and expressed as the mean ± standard error. The probability level was interpreted as statistically significant when the *p*-value was <0.05.

## 3. Results and Discussion

### 3.1. HSSP Treatment of Floating Cells of C. Jejuni

At 0.1 and 0.2 mg/mL concentrations, the number of viable floating *C. jejuni* cells decreased by two and three orders of magnitude, respectively, after HSSP treatment of 1 min (Table 1). Similarly, a previous study [8] reported that treatment with HSSP (0.2 mg/mL) for 1 min reduced the number of floating cells of *E. coli*, *S.* Typhimurium, *Bacillus subtilis*, and *S. aureus* by approximately one order or less magnitude. This indicated that *C. jejuni* was significantly more sensitive to HSSP treatment than other bacteria.

### 3.2. Formation of C. jejuni Biofilms

Lu et al. [32] presented a method for preparing *C. jejuni* biofilms using filter paper. This method allows *C. jejuni* to pass through the filter paper and form colonies. Their study proved that the colonies on paper were biofilms by detecting biofilm components, such as EPS and nucleic acids, by mapping using Fourier transform infrared spectroscopy and Raman spectroscopy [32].

**Figure 1:**
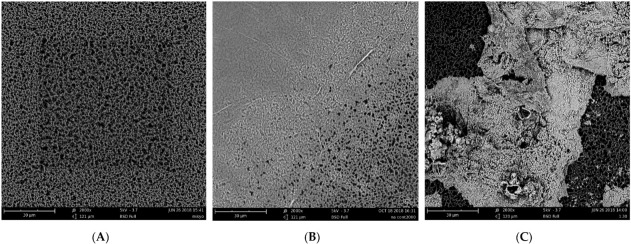
Scanning electron micrographs of *C. jejuni* biofilms on filter paper subjected to HSSP treatment (×2000, bars: 30 µm). (**A**) intact filter paper, (**B**) *C. jejuni* biofilms formed on the filter paper, (**C**) filter paper after HSSP treatment at 10 mg/mL for 2 min.

Figure 1A shows the SEM-based observation of the filter paper in the initial state, in which the pores can be recognized. Figure 1B shows the biofilms observed in these samples. The cells appeared to be connected to the extracellular substance, and the pores of the filter paper were covered. Joshua et al. [33] reported a similar observation in *C. jejuni* biofilms. A portion of the biofilms on the sample was gently scraped off using a loop and Gram stained. Microscopic observation of this specimen confirmed the presence of spiral bacilli (data not shown), implying that *C. jejuni* migrated from the agar surface to the filter paper surface through the pores, proliferated, and formed biofilms.

### 3.3. HSSP Treatment of C. jejuni Biofilm 

HSSP treatment enabled the eradication of *C. jejuni* biofilms (Table 2). At 0.1 mg/mL, HSSP treatment did not completely inactivate biofilms after 30 min. With an increase in HSSP concentration, antibiofilm activity also increased. The number of viable cells in the biofilms was below the detection limit at 1.0 mg/mL for 30 min, 10 mg/mL for 2 min, and 100 mg/mL for less than 1 min. As depicted in Figure 1C, the biofilm sample treated with 10 mg/mL HSSP for 2 min had no biofilms remaining on the filter paper, and the remaining HSSP and exposed filter paper surface could be observed.

*C. jejuni* cells in the biofilm state showed a significant increase in their resistance to HSSP treatment compared with the floating cells of *C. jejuni* (Table 1). Although the sensitivity of floating *C. jejuni* cells to HSSP treatment was markedly higher than that of other bacteria, *C. jejuni* in the biofilm state exhibited almost the same resistance as that of other bacteria in the biofilm state, as reported in previous studies. In the present study, HSSP treatment at 1.0 mg/mL for 20 min decreased the number of viable cells in *C. jejuni* biofilms by approximately four orders of magnitude. Previous studies have shown that under the same conditions, the number of *E. coli*, *S.* Typhimurium, and *S. aureus* in the biofilm state was reduced by approximately four, five, and three orders of magnitude, respectively [24,25,26].

The antibiofilm activity of HSSP was higher than that of NaOH at the same pH (Table 3). In the case of HSSP, the primary reason for its antibacterial activity could be the alkaline effect caused by hydration of the major component CaO. There are two possible reasons for the high antimicrobial efficacy of CaO: the pH of the thin water layer that forms around the particles is much higher than that of the equilibrated solution [34], and active oxygen species, such as superoxide anions, are generated from the HSSP [24] and its main component CaO [35].

*C. jejuni* is a bacterium that causes food poisoning, and there is a need to reduce its presence in the hygiene management of each stage of the food chain, including raw materials, processing, distribution, and cooking of food. This study demonstrated that HSSP treatment could kill and eradicate *C. jejuni* biofilms, suggesting that it is an effective method for controlling *C. jejuni* biofilms.

No negative results were obtained from the sensory evaluation of foods such as fresh vegetables [18,36] and chicken [37] treated with heated shell powder. The addition of heated shell powder prolongs shelf life and improves the sensory and biological quality of kimchi, a traditional fermented vegetable food in Korea, for preservation and consumption [38]. Meat and fish treated with heated shell powder showed less weight loss owing to subsequent heat treatment, and the sensory evaluation yielded satisfactory results. It is not expected that severe problems will be encountered in foods subjected to HSSP treatment [37].

## 4. Conclusions

*Campylobacter* spp. cause food poisoning; hence, measures must be taken to reduce their presence in the hygiene management of each stage of the food chain, including raw materials, processing, distribution, and cooking of food. HSSP is effective in controlling floating cells and *C. jejuni* biofilms. In particular, treatment with more than 10 mg/mL of HSSP could rapidly remove the *C. jejuni* biofilm formed on the filter paper. This effect was significantly greater than that of alkaline treatment (NaOH) at the same pH, indicating that HSSP has a factor other than pH involved in its antibiofilm mechanism. These results suggest that HSSP treatment is an effective means of controlling *C. jejuni* biofilm formation. We are currently investigating the preservation effect of HSSP on chicken meat and its killing effect when inoculated with food-poisoning bacteria.

## Figures and Tables

**Table 1:** Antibacterial effects of heated scallop-shell powder (HSSP) on *Campylobacter jejuni* ATCC 29428 in planktonic cells.

Treatment Time (s)	Survival (log_10_ CFU/mL) *			
	HSSP 0.01 mg/mL	HSSP 0.1 mg/mL	HSSP 0.2 mg/mL	HSSP 1.0 mg/mL
	pH 10.7	pH 11.5	pH 11.8	pH 12.4
0	5.0 ± 0.7 ^a^	5.1 ± 0.7 ^a^	5.4 ± 0.5 ^a^	6.4 ± 0.4 ^a^
30	4.6 ± 0.9 ^a^	3.3 ± 1.3 ^ab^	3.1 ± 0.3 ^b^	ND ^b^
60	4.5 ± 1.0 ^a^	3.5 ± 1.1 ^ab^	2.4 ± 0.4 ^b^	ND ^b^
90	4.5 ± 1.0 ^a^	3.3 ± 1.1^ab^	ND ^c^	ND ^b^
120	4.5 ± 0.9 ^a^	2.8 ± 1.4 ^b^	ND ^c^	ND ^b^

ND: below the detection limit (<2 log_10_ CFU/mL). Statistical analysis for ND was performed using the detection limit (2 log_10_ CFU/mL). * Different letters within columns indicate significant differences (*p* < 0.05).

**Table 2:** Antibiofilm effects of HSSP on the biofilms of *C. jejuni* ATCC 29428 on filter paper.

Treatment Time (min)	Survival (log_10_ CFU/mL) *				
	HSSP 0 mg/mL	HSSP 0.1 mg/mL	HSSP 1.0 mg/mL	HSSP 10 mg/mL	HSSP 100 mg/mL
	Sterilized Water	pH 11.5	pH 12.4	pH 12.8	pH 12.8
0	6.5 ± 0.0 ^a^	6.2 ± 0.2 ^a^	6.2 ± 0.0 ^a^	6.2 ± 0.0 ^a^	6.2 ± 0.0 ^a^
1	−	−	−	3.0 ± 1.5 ^b^	ND ^b^
2	−	−	−	ND ^c^	ND ^b^
3	−	−	−	ND ^c^	ND ^b^
4	−	−	−	ND ^c^	ND ^b^
5	6.4 ± 0.1 ^a^	6.1 ± 0.0 ^a^	4.9 ± 0.8 ^ab^	−	−
10	−	5.4 ± 0.2 ^a^	3.4 ± 0.8 ^b^	−	−
20	−	5.0 ± 0.1^b^	1.8 ± 0.8 ^c^	−	−
30	6.4 ± 0.0 ^a^	3.6 ± 0.9 ^c^	ND ^d^	−	−

ND: below the detection limit (<2 log_10_ CFU/mL). Statistical analysis for ND was performed using the detection limit (2 log_10_ CFU/mL). −: Not performed. * Different letters within columns indicate significant differences (*p* < 0.05).

**Table 3:** Antibiofilm effects of alkaline treatment (NaOH) on the biofilms of *C. jejuni* ATCC 29428 on filter paper.

Treatment Time (min)	Survival (log_10_ CFU/mL) *	
	pH 12.4	pH 12.8
0	6.2 ± 0.0 ^a^	6.5 ± 0.3 ^a^
4	−	5.1 ± 0.1 ^b^
5	4.5 ± 0.2 ^b^	4.6 ± 0.1 ^c^
10	3.9 ± 0.1 ^c^	ND ^d^
20	3.0 ± 0.6 ^c^	ND ^d^
30	3.4 ± 0.0 ^c^	ND ^d^

ND: below detection limit (<2 log_10_ CFU/mL). −: Not performed. * Different letters within columns indicate significant differences (*p* < 0.05).

## Data Availability

Not applicable.

## Abbreviations

CaO	Calcium oxide
CaCO_3_	Calcium carbonate
CFU	Colony-forming unit
*C. jejuni*	*Campylobacter jejuni*
CCDA	Cefoperazone deoxycholate agar
*E. coli*	*Escherichia coli*
HSSP	Heated scallop-shell powder
NaOH	Sodium hydroxide
SEM	Scanning electron microscope
*S.* Typhimurium	*Salmonella* Typhimurium
*S. aureus*	*Staphylococcus aureus*
